# A two-site, two-arm, 34-week, double-blind, parallel-group, randomized controlled trial of reduced nicotine cigarettes in smokers with mood and/or anxiety disorders: trial design and protocol

**DOI:** 10.1186/s12889-016-3946-4

**Published:** 2017-01-19

**Authors:** Sophia I. Allen, Jonathan Foulds, Gladys N. Pachas, Susan Veldheer, Corinne Cather, Nour Azzouz, Shari Hrabovsky, Ahmad Hameed, Jessica Yingst, Erin Hammett, Jennifer Modesto, Nicolle M. Krebs, Junjia Zhu, Jason Liao, Joshua E. Muscat, John Richie, A. Eden Evins

**Affiliations:** 1 Department of Public Health Sciences, Tobacco Center of Regulatory Science, Pennsylvania State University College of Medicine, MC CH69, 500 University Drive, P.O. Box 850, Hershey, PA 17033 USA; 2 Department of Psychiatry, Pennsylvania State University College of Medicine, Hershey, PA USA; 3 Center for Addiction Medicine, Department of Psychiatry, Massachusetts General Hospital, Boston, MA USA; 4 Harvard Medical School, Boston, MA USA

**Keywords:** Tobacco, Cigarettes, RCT, Mood, Anxiety, Spectrum, Cotinine, NNAL

## Abstract

**Background:**

The U.S. Food and Drug Administration can set standards for cigarettes that could include reducing their nicotine content. Such a standard should improve public health without causing unintended serious consequences for sub-populations. This study evaluates the effect of progressive nicotine reduction in cigarettes on smoking behavior, toxicant exposure, and psychiatric symptoms in smokers with comorbid mood and/or anxiety disorders using a two-site, two-arm, double-blind, parallel group, randomized controlled trial (RCT) in four phases over 34 weeks.

**Methods:**

Adult smokers (*N =* 200) of 5 or more cigarettes per day will be randomized across two sites (Penn State and Massachusetts General). Participants must have not had a quit attempt in the prior month, nor be planning to quit in the next 6 months, meet criteria for a current or lifetime unipolar mood and/or anxiety disorder based on the structured Mini-International Neuropsychiatric Interview, and must not have an unstable medical or psychiatric condition. After a week of smoking their own cigarettes, participants receive two weeks of Spectrum research cigarettes with usual nicotine content (11.6 mg). After this baseline period, participants will be randomly assigned to continue smoking Spectrum research cigarettes that contain either (a) Usual Nicotine Content (11.6 mg); or (b) Reduced Nicotine Content: the nicotine content per cigarette is progressively reduced from approximately 11.6 mg to 0.2 mg in five steps over 18 weeks. At the end of the randomization phase, participants will be offered the choice to either (a) quit smoking with assistance, (b) continue smoking free research cigarettes, or (c) return to purchasing their own cigarettes, for the final 12 weeks of the study. The primary outcome measure is blood cotinine; key secondary outcomes are: exhaled carbon monoxide, urinary total NNAL- 4-(methylnitrosamino)-1-(3-pyridyl)-1-butanol and 1-hydroxypyrene, oxidative stress biomarkers including 8-isoprostanes, measures of psychiatric symptoms (e.g., depression, anxiety), smoking behavior and dependence (e.g., cigarette consumption, quit attempts), and health effects (e.g., blood pressure, respiratory symptoms).

**Discussion:**

Results from this study will inform FDA on the potential effects of regulating the nicotine content of cigarettes and help determine whether smokers with mood and/or anxiety disorders can safely transition to significantly reduced nicotine content cigarettes.

**Trial registration:**

TRN: NCT01928758, registered August 21, 2013.

## Background

Tobacco smoking is the leading preventable cause of premature morbidity and mortality in the U.S., [1] and cessation proffers immediate and sustained improvement in health and quality of life [2]. The Family Smoking Prevention and Tobacco Control Act [3] gave the U.S. Food and Drug Administration (FDA) jurisdiction to regulate tobacco products, including the nicotine content of cigarettes. Progressive reduction of the nicotine content of cigarettes to very low levels is a potential way to reduce the addictiveness of cigarettes, and may make it easier for established smokers to quit smoking [4–6]. Preliminary studies have found that progressive reduction of nicotine content of cigarettes is feasible and safe in smokers without comorbid psychiatric illness [5, 7–10]. However, it is not known whether progressive nicotine reduction is feasible and safe in the large subgroup of smokers with comorbid psychiatric illness.

Smokers with psychiatric illnesses purchase over 40% of cigarettes sold in the U.S., [11] have a higher prevalence of smoking, greater severity of nicotine dependence and lower cessation rates than smokers without comorbid psychiatric illness [12, 13]. Smokers with a prior mood or anxiety disorder report more severe nicotine withdrawal symptoms during a cessation attempt [14, 15]. This suggests that a policy to reduce nicotine content in cigarettes may differentially impact smokers with affective disorders such that they may have more severe nicotine withdrawal symptoms and, as a result, may smoke a greater number of low nicotine cigarettes in order to reduce withdrawal symptoms, thereby increasing their exposure to other toxicants in tobacco smoke, a process termed compensatory smoking [16, 17]. On the other hand, smokers who transition to significantly reduced nicotine content cigarettes may be more likely to quit smoking due to a lower level of nicotine dependence. This study aims to test both these hypotheses.

The impact of reduced nicotine cigarettes on smoking behavior, toxicant exposure and cessation needs to be assessed empirically prior to implementation of a national nicotine reduction policy [4, 18]. Early studies of the behavioral effects of a progressive nicotine reduction strategy have been encouraging. A series of studies by Benowitz and colleagues examined the effects of a progressive nicotine reduction strategy in which smokers switched to cigarettes with progressively lower nicotine content (or yield) in 6 steps, over 10 weeks or over 6 months [7–9]. In their initial study of 12 smokers, reduced nicotine content (RNC) cigarettes were found to be a feasible option for reducing nicotine addiction [9]. In a subsequent study, 20 smokers consumed gradually reduced nicotine content cigarettes which did not increase apparent exposure to tobacco smoke toxins [7]. In a follow up study, 135 otherwise healthy smokers were randomly assigned to one of two groups: (a) an experimental group that smoked their usual brand for two weeks followed by research cigarettes with five levels of progressively reduced nicotine content (first four levels for 4 weeks each and the fifth level for 6 months), or (b) a control group that continued smoking their own brand of cigarettes for the entire study period [8]. In the smokers assigned to RNC cigarettes, nicotine intake, as assessed by plasma cotinine concentration, declined progressively as the nicotine content of the cigarettes was reduced, while toxin exposure remained stable or, in the case of the carcinogen, (4-(methylnitrosamino)-1-(3-pyridyl)-1-butanol) (NNAL), was reduced [8]. The titration was well tolerated, but smokers assigned to the RNC group reported decreased “vigor” scores and increased “confusion” scores on the Profile of Mood States (POMS) during the time they were smoking 2 mg and 1 mg nicotine content cigarettes (compared with baseline), and smokers assigned to the RNC group who were adherent to study procedures also had a 2 kg increase in body weight during the study period [8]. These effects are consistent with nicotine withdrawal symptoms [19]. Significantly more of the RNC group (51%) than the control group (14%), were interested in quitting smoking by the end of the tapering period. In a recent study, Donny and colleagues [10] conducted a 6-week, double-blind, multi-site, parallel, randomized controlled trial among 840 smokers who were either assigned to smoke (1) their usual brand cigarettes (study cigarettes), (2) investigational cigarettes with nicotine content similar to commercial products (primary control cigarettes), or (3) one of five investigational cigarettes with 2 to 33% of the nicotine in the primary control cigarettes. Overall, participants assigned to the lowest nicotine content cigarettes significantly reduced the number of cigarettes smoked per day, which resulted in minimal compensation, reduced nicotine exposure and dependence, and were more likely to report quit attempts after a 30-day period following the six-week trial compared to those assigned to the primary control cigarettes [10]. These studies among others [5, 20, 21] demonstrate that a progressive nicotine reduction strategy is feasible, results in reduced nicotine exposure, increases desire to quit smoking and does not result in increased exposure to other toxicants.

These studies have been well conducted, clear in their conclusions and represent a critical first step in the process of empirically evaluating the effects, intended or unintended, of a policy of progressive nicotine reduction in cigarettes. However, there are some issues regarding generalizability of the results that must be evaluated prior to implementation of such a policy. A large proportion (>40%) of cigarettes sold in the United States are sold to those with a current psychiatric illness, and smoking prevalence is demonstrably higher among those groups [11]. However, enrollment in the studies conducted to date has focused on relatively well-educated, otherwise healthy smokers. In one study, 35% of those screened were excluded for current drug or alcohol dependence and 20% for other health issues, and those enrolled had an average of 15 years education [8]. Further, there was differential drop out for those with more severe nicotine dependence as evidenced by higher baseline Fagerström Test for Nicotine Dependence (FTND) scores. In more recent studies [10, 22], participants were excluded for having a serious medical or psychiatric disorder, other than depression.

Studies of potentially modified risk tobacco products to date have measured a range of biomarkers, but have tended to focus on certain reliable key measures of nicotine exposure (e.g. cotinine), smoke exposure (e.g. exhaled carbon monoxide [CO]), and carcinogen exposure (e.g. NNAL). These markers have the advantages of being well validated, are relatively specific to tobacco smoking, and change relatively quickly in response to changes in exposure. However, tobacco products are a major cause of oxidative stress and evaluation of the potential risks of different tobacco products should include measurement of biomarkers of oxidative stress. Tobacco smoke is an abundant source of free radicals, containing over 10^17^ reactive oxygen and nitrogen species (ROS/RNS) per puff and considerable evidence indicates that these agents play fundamental roles in the development of many of the major smoking-caused diseases including cancer, chronic obstructive pulmonary disease (COPD) and heart disease [1]. This study will therefore include measures of oxidative stress at baseline and during the randomized portion of our proposed trial. We believe this will be the first time that valid biomarkers of oxidative stress will be assessed in a randomized trial of RNC cigarettes.

### Aim

The specific aims are to assess the effect of switching to gradually reduced nicotine content cigarettes on:

1. Product use patterns and biomarkers of exposure in smokers with unipolar mood and/or anxiety disorders

We will assess biomarkers of exposure including blood cotinine (primary measure), exhaled CO, and urinary total NNAL and 1-hydroxypyrene. In addition, oxidative stress biomarkers will be measured including 8-isoprostanes. We hypothesize that smokers assigned to the RNC group will have lower plasma cotinine concentrations during the last 6 weeks of the randomized phase of the study than those assigned to the usual nicotine content (UNC) group. We further hypothesize that there will be no significant increase in cigarette consumption, nicotine-independent biomarkers of tobacco use (e.g., 1-hydroxypyrene, exhaled CO), or adverse health effects (e.g., high blood pressure, adverse respiratory symptoms) in those assigned to the RNC group vs. those assigned to the UNC group.

2. Psychiatric and nicotine withdrawal symptoms in smokers with unipolar mood and/or anxiety disorders

We will measure psychiatric and nicotine withdrawal symptoms using the Quick Inventory of Depressive Symptomatology (QIDS) [23], Overall Anxiety Severity and Impairment Scale (OASIS) [24], Minnesota Nicotine Withdrawal Scale (MNWS) [25], and other measures of stress/mental health. We hypothesize there will be no significant increase in ratings of psychiatric or nicotine withdrawal symptoms in those assigned to the RNC group as compared to those assigned to the UNC.

3. Self-perception of tobacco dependence, self-report of intention to quit smoking, and actual smoking cessation attempts

We hypothesize that smokers assigned to the RNC group will have lower perceived dependence, be more likely to report intention to quit smoking in the next six months, and be more likely to make a smoking cessation attempt during the study.

## Methods/Design

This is a two-site, two-arm, double-blind, parallel group, randomized controlled trial that will proceed in four phases over 34 weeks. The two study sites are Penn State University (PSU) College of Medicine and Massachusetts General Hospital (Mass. General). Participants and study staff will be blind to the experimental cigarette allocation from the randomization visit to the last visit. Visits will occur at consistent times during the day.

### Blinding of the research cigarettes

Cigarette cartons will arrive in the Penn State Investigational Drug Service (IDS) with a packaging slip that provides information about the cartons in the shipment which includes the nicotine content, carton bar code number and a batch/lot number. This information will be carefully recorded by the Cigarette Manager into our Cigarette Management System (CMS), removed from the carton and replaced with a blind code number. Individual packs do not contain any identifiable information. Each individual pack will be labeled with the carton blind code.

### Study population

Tobacco cigarette smokers with a history of unipolar mood and/or anxiety disorders throughout the greater Hershey, Pennsylvania and Boston, Massachusetts areas who report no quit attempt in the past month and no plan to quit smoking in the next 6 months will be recruited to the trial.

### Inclusion and exclusion criteria

The study inclusion criteria are as follows:

- aged 18–65
- planning to live in the local area for the next 8 months
- report smoking >4 cigarettes per day (regular filtered cigarettes or machine-rolled cigarettes with a filter) for at least the past 12 months
- no quit attempt in the prior month and not planning to quit smoking in the next 6 months
- no use of varenicline, bupropion (used specifically as a quitting aid); nicotine patch; gum; lozenge; inhaler; or nasal spray in prior month
- meet lifetime diagnostic criteria for a current or lifetime unipolar mood disorder (dysthymia, major or minor depression, premenstrual dysphoric disorder) or anxiety disorder (panic disorder, obsessive-compulsive disorder; post-traumatic stress disorder; mixed anxiety depressive disorder, agoraphobia, generalized anxiety disorder, social phobia, specific phobia) based on the Mini-International Neuropsychiatric Interview, MINI [26]
- ability to read and write in English, comprehend and consent to study procedures is required

The study exclusion criteria are listed below:

- pregnant and/or nursing
- any unstable or significant medical conditions such as elevated blood pressure (systolic >160 mmHg at baseline), recent heart attack or some other heart condition, stroke, or severe angina, COPD requiring oxygen, use of oral prednisone, kidney (e.g., dialysis) or liver diseases (e.g., cirrhosis)
- any medical disorder/medication that may affect participant safety or biomarker data
- use of any non-cigarette nicotine delivery product (e.g., cigar, pipe, chew, snus, dip, hookah, electronic cigarette, strips, sticks) in the past 7 days
- other serious mental illness (e.g., schizophrenia, bipolar disorder, current eating disorder, and dementia) or any inpatient psychiatric or substance abuse treatment in the past 6 months
- current suicide risk on clinical assessment (above “low risk” score on MINI [26] diagnostic interview)
- weekly use in the past 3 months of illegal drugs or prescription drugs that are not being used for medically prescribed purposes
- alcohol use that would hinder the participant’s ability to participate
- a history of difficulty providing or unwilling to provide blood samples (e.g., fainting, poor veins)
- surgery requiring general anesthesia in the past 6 weeks
- unwilling to remain on one flavor of research cigarette (regular or menthol) for the duration of the trial or smokes hand-rolled cigarettes
- another member of household participated or currently participating in the study
- prisoners (at the time of enrollment)
- any other condition or situation that would, in the investigator’s opinion, make it unlikely that the participant could comply with the study protocol

### Early withdrawal of subjects

This study is designed to identify participants who are not able or unwilling to comply with the full study protocol during two baseline phases (I and II) prior to randomization. During Baseline I, participants will smoke their usual brand of cigarettes for one week. At Baseline II, all participants will be asked to smoke Spectrum research cigarettes [27] with a usual nicotine content (about 11.6 mg) for two weeks. Participants who are removed prior to randomization will be replaced until a total of 200 participants have been randomized.

### Recruitment, consent process and documentation

Participants will be recruited at both sites throughout the Hershey and Boston areas by using media advertisements (newspaper, radio, internet); study posters and flyers placed on community message boards, in local businesses, and in clinics; community newsletters, social media sites (e.g., Facebook) and internet websites (e.g., Craigslist). Interested volunteers who call the study center number will first complete basic eligibility questions over the phone. After meeting eligibility criteria over the phone, participants will be scheduled to come into the study center where they will be consented to the study and further screened and assessed for eligibility.

### Procedures

#### Phase I: use of usual cigarette brand (baseline i – one week)

During Visit 1 screening (in-person), informed consent will be obtained from the participant by study staff and the usual discussion of procedure, risks, side effects, confidentiality, voluntary participation, and right to refuse participation without prejudice will be explained to the participant. Participants must be capable of understanding the nature of this study, its potential risks, discomforts and benefits before signing consent. After consent is obtained, study staff will administer the MINI [26], screen for drug abuse, obtain medical and concomitant medication histories, and measure vital signs (e.g., blood pressure and heart rate), with eligibility determined based on inclusion/exclusion criteria previously mentioned. For women of child bearing status, a urine sample will be collected and eligibility screening will include a pregnancy test.

Once a participant has been determined to be eligible, biomeasures will be obtained; specifically, exhaled CO, lung function (spirometry), height, weight, and waist and hip circumferences. Participants will be asked to complete questionnaires (see Table 1) and study staff will review the study guidelines and provide participants with instructions on how to keep track of the number of their usual brand cigarettes smoked each day for one week by using a cigarette log. All participants will be asked to refrain from using other tobacco products or illegal drugs for the remainder of the study, but are encouraged to report the use of these products to the study staff.

**Table 1:** Time and events schedule with measures, questionnaires, and procedures for study

	Baseline				Randomization						Treatment Choice	
	Baseline I		Baseline II		Step 1	Step 2	Step 3	Step 4	Step 5			
Study Week Number	0	1	2	3	6	9	12	15	18	21	25	33
Study Day	1	8	15	22	43	64	85	106	127	148	176	232
Study Visit Number	1	2	3	4	5	6	7	8	9	10	11	12
Measures/Questionnaires												
Tobacco and marijuana use and daily cigarette log^a^		X	X	X	X	X	X	X	X	X	X	X
Concomitant medications^a^		X	X	X	X	X	X	X	X	X	X	X
Adverse events^a^ [39]		X	X	X	X	X	X	X	X	X	X	X
Demographics	X											
Tobacco use history, cigarette details^a^	X	X										
NIDA drug use (past 3 mo) [40]	X						X			X		
Environmental smoke questionnaire [41]		X		X	X	X	X	X	X	X	X	X
Perceived Health Risk		X		X			X			X		
Cigarette Liking Scale [42]	X	X	X	X	X	X	X	X	X	X		
Nicotine dependence (HONC, FTND, PSU) [43–45]	X	X	X	X	X	X	X	X	X	X	X	X
Minnesota Nicotine Withdrawal Scale [25]	X	X	X	X	X	X	X	X	X	X	X	X
Smoking urges [46, 47]	X	X		X	X	X	X	X	X	X	X	X
Audit-C [48, 49]		X		X		X		X		X		
Anxiety symptoms questionnaire (ASQ) [50, 51]	X						X			X	X	X
CES-D[52]	X						X			X	X	X
Kessler K6 scale [53, 54]	X	X		X	X	X	X	X	X	X	X	X
OASIS (anxiety) [24]	X	X		X	X	X	X	X	X	X	X	X
QIDS (depression) [23]	X	X		X	X	X	X	X	X	X	X	X
Perceived Stress Scale [55]	X	X		X	X	X	X	X	X	X	X	X
Clinical COPD questionnaire [56]		X		X	X	X	X	X	X	X	X	X
Menthol [57]		X	X	X	X	X	X	X	X	X	X	
Pittsburgh Sleep Symptom and Dreams Questionnaires [58, 59]	X									X		
INTERHEART and WI-PREPARE [60, 61]	X									X		
Smoking cessation (if chosen by participant) [29]										X	X	X
NRT disbursement (if chosen by participant)										X	X	
Biomeasures/Procedures^b^												
Weight [62]	X	X	X	X	X	X	X	X	X	X	X	X
Height [62]	X											
Waist hip ratio [62]	X									X		
Exhaled CO [63]	X	X	X	X	X	X	X	X	X	X	X	X
Blood pressure/pulse [39, 64]	X	X	X	X	X	X	X	X	X	X	X	X
Pulmonary function (spirometry) [65]	X	X			X			X	X	X	X	X
Urine collection		X		X	X	X	X	X	X	X	X	
Pregnancy test	X			X		X		X		X	X	X
Blood collection for biomarker analysis		X		X	X	X	X	X	X	X	X	
Payment	$40	$80	$40	$80	$80	$80	$80	$80	$80	$80	$80	$40+ compliance

*NIDA* National Institute on Drug Abuse, *HONC* Hooked on Nicotine Checklist, *FTND* Fagerstrom Test for Nicotine Dependence, *PSU* Pennsylvania State University, *ASQ* Anxiety Screening Questionnaire, *CES-D* Center for Epidemiologic Studies Depression Scale, *OASIS* Overall Anxiety Severity and Impairment Scale, *QIDS* Quick Inventory of Depressive Symptomatology, *COPD* chronic obstructive pulmonary disease, *WI-PREPARE* Wisconsin Predicting Patient’s Relapse, *NRT* nicotine replacement therapy

^a^To be completed by the researcher. All others will be completed by the participant

^b^All biomeasures/procedures to be completed by researcher. Some biomarkers will be analyzed in selected subgroups of collected samples

During Visit 2, participants will be asked for their cigarette log from the prior week and to complete questionnaires. Biomeasures similar to Visit 1 will be obtained except, height, waist, and hip measurements. Blood (about 2 teaspoons) and urine samples will be collected at this and future visits (except 3 & 12) for analysis. Participants will be given Spectrum research cigarettes containing a normal amount of nicotine (11.6 mg) matching the flavor (regular or menthol) of their usual brand of cigarettes for one week. The number of research cigarettes provided will be 150% of baseline cigarettes per day and may be increased throughout the study according to recent consumption in order to reduce the chance of running out. Participants will be asked to return all opened, unopened and empty cigarette packs to the study center at each visit. Participants who are accurate with returning their cigarette packs and attend all study visits will be eligible for additional compliance payments at the end of the study.

#### Phase II: use of normal nicotine content research cigarettes (baseline ii – 2 weeks)

At Visit 3 (Baseline II), biomeasures similar to Visit 2 will be obtained except for lung function (spirometry) and no blood or urine will be collected. Participants will be asked to complete questionnaires and will be asked for their cigarette log from the prior week. All participants will continue to smoke Spectrum research cigarettes containing a normal amount of nicotine (11.6 mg) for an additional 1 week.

Visit 4 will be similar to Visit 2 with the same biomeasures, completion of questionnaires, and collection of cigarette logs; however the participants who complete Baseline II and agree to continue will enter the Randomization Phase. They will be randomized to either (1) continue to smoke the same 11.6 mg nicotine Spectrum research cigarettes they smoked in Baseline II for 18 additional weeks (UNC) or (2) switch to progressively reduced nicotine content (RNC) cigarettes over 18 weeks (see Table 2). Participants will receive a 3-week supply of research cigarettes at 150% of baseline daily consumption to last until their next visit. There will be no adjustments outside of the dosing schedule. Participants in the control group will receive the same research cigarettes with the same nicotine content throughout the trial.

**Table 2:** Nicotine content dosing schedule for study

Phase	Baseline I	Baseline II	Randomized Double-Blind Phase					Treatment Choice Phase
Week(s)	1	2	3	3	3	3	6	12
Cigarette type	Own Brand	Usual Nicotine Research Cigarettes	Reduced Nicotine Step 1	Reduced Nicotine Step 2	Reduced Nicotine Step 3	Reduced Nicotine Step 4	Reduced Nicotine Step 5	Variable
Spectrum Code: regular		600	500	400	300	200	102	
Spectrum Code: menthol		601	501	401	301	201	103	
Approximate nicotine content in mgs per cigarette (mg/gram)^a^								
RNC	Around 13 mg^b^	11.6	7.4	3.3	1.4	0.7	0.2	Variable
	(19)	16.5	(10.6)	(4.7)	(1.9)	(0.9)	(0.3)	
UNC	Around 13 mg^b^	11.6	11.6	11.6	11.6	11.6	11.6	Variable
	(19)	(16.5)	(16.5)	(16.5)	(16.5)	(16.5)	(16.5)	

^*a*^These are averages of menthol/non-menthol cigarettes at each level based on estimated 0.7 g tobacco content per cigarette and nicotine concentrations based on Richter et al. [27]

^b^Estimated mean nicotine content and concentration based on Connolly et al. [66]

#### Phase III: use of randomly assigned nicotine content cigarettes (18 weeks)

During Phase III (Randomization), participants will have weekly contact with the study center staff, including a visit every three weeks. One week after an in-person study visit, participants will complete surveys either over the phone or by clicking on a secure survey link sent to their personal email account. Two weeks following the in-person visit (one week after email survey/phone contact) the study center staff will contact participants to check on progress and ask questions about the cigarettes being smoked. Three weeks following the in-person visit, participants will visit the study center to complete questionnaires, and return all open, unopened and empty cigarette packs at each visit during this Phase. They will be given another 3-week supply of research cigarettes and biomeasures will be collected through Visit 11 as listed in Table 1.

During Visit 10, the last visit of the Randomization Phase, participants will be given the choice on how they would like to complete the rest of the study and will be encouraged to quit smoking. All participants will receive a copy of the U.S. Surgeon General Report, “How Tobacco Causes Disease” [28] and resources available in the community to help smokers quit. Choices that will be provided to the participant will include:

1. Return to their usual brand of cigarettes for 12 weeks (purchased at their own cost).
2. Continue to receive the research cigarettes for 12 weeks (provided at no cost).
3. Quit smoking with brief counseling from the study team and the option to use oral nicotine replacement therapy (NRT [gum or lozenge]) for 11 weeks.

If participants choose to return to their usual brand of cigarettes, they will not be given any more research cigarettes and will be removed from the CMS, given cigarette pack paper logs and instructed to bring all logs back to the next study visit 4 weeks later. If participants choose to continue on research cigarettes, they will be given a 4-week supply of either UNC or RNC research cigarettes (150% of baseline cigarettes per day) corresponding to the cigarettes they were given at the last visit of the participant’s treatment group allocation, given cigarette pack paper logs and instructed to bring all logs and research cigarette packs back to the next study visit 4 weeks later. If participants choose to quit smoking, they will be given up to a 6-day supply of research cigarettes corresponding to the cigarettes they were given at the last visit of the participant’s treatment group allocation. These cigarettes are intended to last until their target quit date. Participants who choose to quit will also be given cigarette pack paper logs and instructed to bring all logs and research cigarette packs back to the study center one week later for their in-person counseling session. Regardless of the participant’s choice, all participants will attend two additional follow up visits four weeks (visit 11) and 12 weeks (visit 12) after the end of the randomized phase.

#### Phase IV: treatment choice (12 weeks)

##### Quit arm

Participants who choose to quit smoking must be willing to set a quit date within the following week and will be offered a flexible smoking cessation treatment. They will have the option to receive up to 11 weeks of short-acting NRT (gum or lozenges) at no cost and cognitive behavioral-based smoking cessation counseling provided by study staff, in-person or over the phone. In addition to regular study visits (week 25 [Visit 11] and week 34 [Visit 12]), there will be two optional additional in-person sessions (weeks 23 and 30), four phone sessions (weeks 23, 24, 28, and 32) and five self-guided sessions. In order to avoid missed phone sessions, researcher and participant will attempt to agree on a standing appointment, day and time. Participants will receive 20 min (or less) of standard individual cognitive behavioral therapy (CBT) based on the *Freedom from Smoking* (FFS) curriculum (http://www.ffsonline.org/) from the American Lung Association. They will be given strategies to cope with triggers/urges to quit.

During the first in-person quit visit (week 22), one day after the participant’s target quit date, exhaled CO will be measured, adverse events and concomitant medications will be assessed and participants will complete questionnaires. Participants will return all cigarette packs (6-day supply), bring completed cigarette logs, and be evaluated by study staff for nicotine withdrawal symptoms and be removed from the CMS. If the participant chooses to use NRT they will receive 3 boxes of either nicotine gum (110 pieces/box) or lozenges (81 pieces/box) according to the participant’s preference. Adverse events and concomitant medications will be assessed and a questionnaire (Smoking Cessation Quit Day) [29] that includes the MNWS [25]. By this visit, some participants will have transitioned to smoking very low nicotine content research cigarettes. Decisions about dosing will be determined based on participant reported withdrawal symptoms. Participants who may have tapered to very low nicotine cigarettes should have minimal withdrawal symptoms and may require very low NRT dosing. A general guideline for NRT dosing will be used to make recommendations to participants as follows:

Group 1:

- Not able to remain abstinent or
- Reports a score of 2 or more on the MNWS items #1 and #4 (irritable/angry and/or craving to smoke)

Group 2:

- Able to remain abstinent and
- Reported a score of 0 or 1 on MNWS items #1 and #4 or
- Had few slips

The day after the week 22 visit, a researcher will call the participant to assess whether the participant is experiencing any side effects from the NRT. Additional courses of NRT will be given to the participant, as needed, either at study visits, or if the participant calls in to the study center to request additional NRT.

At Visits 11 and 12 (Treatment Choice), all participants will complete questionnaires and study procedures as done in previous visits, except no blood or urine will be collected at Visit 12. At Visit 11, if participants chose to receive research cigarettes, they will be given an additional 8-week supply of either UNC or RNC research cigarettes (150% of baseline cigarettes per day) corresponding to the cigarettes they were given in the last visit of the participant’s treatment group allocation. Participants who chose to use NRT to quit smoking, could be given a refill of oral NRT (3 boxes of gum or lozenge) as appropriate depending on the participant’s NRT usage and withdrawal symptoms. For participants who chose to quit smoking, the NRT dosing schedule will be discussed at weeks 26 and 30.

At Visit 12, no further counseling, NRT or study cigarettes will be given to the participants. The study cigarette log will be reviewed and the total number of cigarettes smoked will be recorded. Each participant will be assessed for eligibility for the final study incentive payments for (a) study visit compliance ($50) and (b) cigarette pack return accuracy ($50). Participants will be eligible for study visit compliance if they attended all study visits and provide data. Participants will be eligible for the cigarette pack return accuracy compliance incentive if they returned all research packs (opened and unopened) within a margin of 4 packs for 6 out of 8 study visits where research cigarette return was required (Visits 3, 4, 5, 6, 7, 8, 9, and 10).

**Fig. 1:**
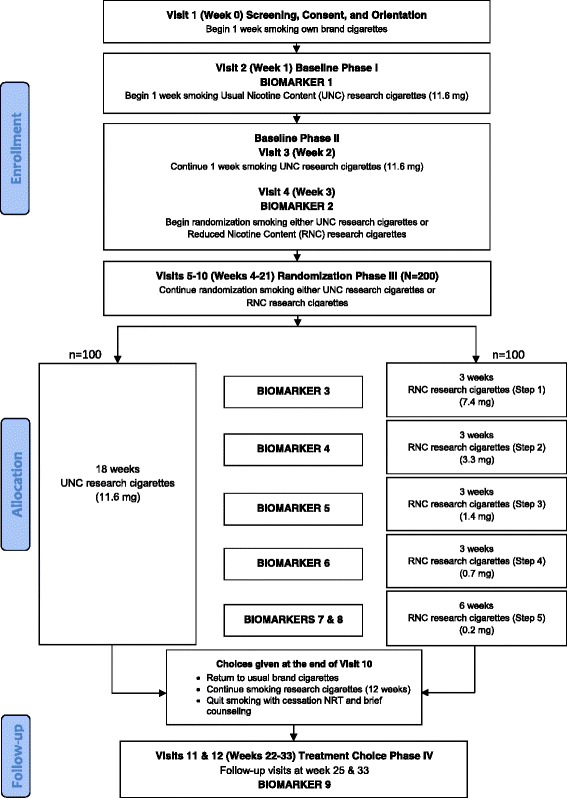
Study design timeline for the two-site, two-arm, 34-week, double-blind, parallel group, RCT in four phases

The sequence for all study visits, from screening to follow-up are shown in Fig. 1.

#### Primary outcome

The primary outcome variable is plasma cotinine concentration (measured as ng/ml) during the last 6 weeks of the randomized phase. The mean value of this continuous measurement will be compared between two groups: the experimental group (RNC cigarettes) and the control group (UNC cigarettes).

#### Secondary outcomes

The secondary outcome measures include exhaled CO and urinary total NNAL and 1-hydroxypyrene, cigarette consumption, and health effects (e.g., blood pressure, respiratory symptoms). In addition, oxidative stress biomarkers including 8-isoprostanes will be measured. Some biomarkers will be analyzed in selected subgroups of collected samples.

The proportion of each group making a choice to try to quit smoking will be an endpoint, as will the proportion succeeding in quitting successfully during the choice phase. Intent to treat (ITT) abstinence (based on the assumption that a loss-to-follow-up subject resumes smoking) and completers’ abstinence (based on the assumption that the probability of loss to follow up is independent of smoking status) will be analyzed. Abstinence will be biochemically verified (CO ≤9 ppm).

#### Sample size

For plausible effect size and variation, we used results of Benowitz et al. [8], where the mean and standard deviation of plasma cotinine concentration are 240 and 120 for the control group and 113 and 116 for the experimental group at the 22nd week follow-up visit (end of randomization phase for trial). It is expected that the cotinine level in the experimental group will reduce gradually after start of the trial and the mean difference of the cotinine level between these two groups for our study will be smaller than the difference of 127 ng/ml in Benowitz et al. [8]. With a sample size of 70 participants per group, we are able to detect the difference in plasma cotinine concentration level between the two groups as small as 58 ng/ml (which could occur on the 7.4 mg nicotine content cigarettes) with at least 80% power (and 68 ng/ml with at least 90% power). Allowing for a 30% participant –withdraw rate, we plan to recruit 100 participants for each group (a total of 200). The alpha level used for the power analysis is 0.05.

Additional statistical power analyses will be performed based on secondary aims: to examine the difference in biomarkers of tobacco smoke exposure, nicotine withdrawal, and psychiatric symptomatology, with a sample size of 70 subject completers per group, we are powered to detect an effect size of 0.5 (difference is half of the standard deviation) between the two groups with at least 83% power. Also, with a completing sample size of 70 per group we are able to detect a 15-20% difference in the proportion of subjects willing to quit between these two groups (depending on the proportion in the control group) with about 80% power. If the proportion difference was as large as the value in the Benowitz et al. [8] study (51% of the RNC group versus 14% of the control group) then our statistical power will be as high as 99.8%. It was assumed that both groups have equal drop-out rate (30%) when the sample size was calculated above. However, it is possible that we could have differential drop­out rate in the two groups. Benowitz, et al. [8] had 9% dropout in the control group versus 33% in the intervention group. We have 99% statistical power in detecting a difference as large as this (9% vs. 33% for dropouts).

#### Withdrawal criteria

Participants will be withdrawn from the study any time prior to randomization if:

1. They report using non-cigarette nicotine products at more than one visit. This includes any number of cigars, pipes, snuff, chew, hookah, electronic cigarette, marijuana, or any other illegal smoked substance.
2. They are not able to attend a study visit within the allowed visit windows (i.e., participant misses a scheduled study visit AND any subsequent make-up visit that is arranged).

During Baseline II only, participants will be withdrawn from the study if:

1. Participant’s total cigarette consumption includes more than 10% of non-research cigarettes in the 6 days prior to visit 4 only (average cigarettes from day 15–20, e.g., 4 or more out of 30 cigarettes in 6 days for a 5 cigarette per day (CPD) smoker; 18 or more out of 180 cigarettes in 6 days for a 30 CPD smoker).
2. Participant has reduced their cigarette consumption by more than 50% from baseline (when CPD are averaged over days 15–20).
3. Significant baseline smoking rate increase: A participant will be withdrawn from the study if they meet BOTH of the following criteria:
  - The average CPD increase by more than 100% from the average CPD at the Baseline II assessment (visit 4) when calculated over the previous 6 days.
  - The average of two consecutive expired breath carbon monoxide (CO) measurements increase according to the following:
    i. CO is greater than 50 ppm if CO at assessment visit 1 is <20 ppm.
    ii. CO is greater than 60 ppm if CO at assessment visit 1 is 20–34 ppm.
    iii. CO is greater than 70 ppm if CO at assessment visit 1 is 35–49 ppm.
    iv. CO is greater than 80 ppm if CO at assessment visit 1 is 50–60 ppm.
    v. CO is greater than 90 ppm if CO at assessment visit 1 is 61–70 ppm.

Participants may be discontinued by the investigator at any point during the study for any reason that meets the inclusion, exclusion, or early withdrawal criteria. If participants are withdrawn from the study for any reasons noted above during Baseline I or II (prior to randomization), they will be replaced until a total of 200 participants have been randomized to the study. Participants who voluntarily withdraw from the study will be asked to complete a questionnaire regarding the reasons for dropping out and what they didn’t/did like about the study.

#### Data management and monitoring

Participants will be under medical supervision while in the study and seen on an ongoing basis by our study staff who will assess adverse events and make appropriate referrals to the physician. The Data Safety Monitoring Board (DSMB) will oversee the safety of the participants in the trial. The DSMB will receive summary reports on recruitment, retention, adverse events (AEs), and CO and produce a report and recommendation annually.

#### Statistical analysis

Study data will be collected and managed using REDCap electronic data capture tools hosted at the Penn State Milton S. Hershey Medical Center and College of Medicine. REDCap is a secure, web-based application designed to support data capture for research studies [30]. Statistical analysis and additional data management will be performed using R 3.3 (R foundation, https://www.r-project.org/). Complete summary statistics will be provided for all variables at baseline and across the trial period, separately for the two trial arms where appropriate. Graphs such as Boxplots and longitudinal trajectory plots will be used to visualize the data. Outliers and questionable data points will be investigated for quality assurance. The demographic characteristics, smoking history and other baseline measures will be compared between the two arms to validate the effectiveness of the randomization procedure.

The statistical analysis will focus on comparing the two trial arms (reduced nicotine content vs. usual nicotine content cigarettes) on the following endpoints: (a) cotinine concentrations during the last 6 weeks of the randomized phase; (b) change of QIDS depression level and OASIS anxiety measure from baseline to last 6 weeks of randomized phase; (c) dropout rate from the trial by the end of the randomized phase; (d) rate of psychiatric serious AEs and (e) the proportion of subjects that try to quit smoking at the end of the randomized phase. For endpoints (a) and (b), mixed effects regression models will be used to model their change over the trial period and the difference of the trajectories between the two arms. For endpoints (c), (d) and (e), logistic regression and Poisson regression will be used. Other endpoints such as smoking behaviors will also be compared but on an exploratory basis.

Compliance, along with dropouts, will be treated as an additional outcome measure. We will conduct a separate statistical analysis for the subgroup of participants who are judged to be completely compliant. Non-compliance will be described as a continuous as well as a categorical measure (greater than 10% of cigarettes smoked were non-study cigarettes). We will also examine the extent of use of pharmaceutical products or other alternative tobacco products during the 18 week randomized study period. In our analysis, we will add both dropouts and compliance as an outcome variable.

## Discussion

Among nicotine dependent smokers, 22% have a mood disorder and 23% have an anxiety disorder (compared with 7% and 10% of nonsmokers) [31]. Smokers with a comorbid psychiatric illness may be more vulnerable to unintended effects of a progressive nicotine reduction policy at the national level. These smokers, by the nature of their comorbid psychiatric illness, may experience chronic high levels of psychological distress, may be more sensitive to nicotine withdrawal symptoms, and may be more likely to exhibit compensatory smoking behavior in order to maintain systemic nicotine concentrations. Smokers with mental illnesses report wanting to quit smoking, trying to quit smoking, and use of smoking cessation aids at similar rates to people without mental health problems, but have higher levels of nicotine dependence and may be less likely to succeed when they attempt to quit smoking than smokers without a comorbid psychiatric illness [32, 33].

The reasons for the high comorbidity between mood and anxiety disorders and nicotine dependence are not fully understood. Many studies have found that smokers with affective disorders report elevated nicotine withdrawal symptoms during early abstinence, compared to those without [15, 34–36]. Potential mechanisms have been identified. For example, smokers with a prior major depressive episode have greater smoking-induced brain dopamine release than those without such a history [37]. Monoamine oxidase A (MAO-A), an enzyme that metabolizes monoamines, including dopamine, and, through this mechanism, modulates mood, is increased in the brains of heavy smokers during abstinence. This effect, which covaries with severity of depression, suggests one possible biological mechanism for depressive symptoms as part of the nicotine withdrawal syndrome [38].

For the reasons described above, smokers with mood and anxiety disorders may be particularly vulnerable to unintended effects of a policy of progressive nicotine reduction in cigarettes. It is not known if smokers with these conditions are more likely to “oversmoke” reduced nicotine cigarettes and experience increased toxin exposure as a result. It is also possible that smokers with mood and anxiety disorders may experience some exacerbation in their psychiatric symptoms as they gradually withdraw from nicotine under a nicotine reduction policy. On the other hand, if smokers with mood and anxiety disorders can safely transition to significantly reduced nicotine content cigarettes, it is plausible that further progression to smoking cessation may be more achievable from a lower level of nicotine dependence. We therefore propose to examine the effects of switching to reduced nicotine content cigarettes in smokers with current anxiety and mood disorders. We see this as a critical test of the feasibility of the RNC regulatory strategy.

Results from this study will inform on effects of regulating nicotine content of cigarettes and help determine whether smokers with mood and anxiety disorders can safely transition to significantly reduced nicotine content cigarettes and further progress to smoking cessation.

## Abbreviations

CO: carbon monoxide
NNAL: 4-(methylnitrosamino)-1-(3-pyridyl)-1-butanol
